# Supplementary material for: Laughter regulation in solitary and social contexts varies across emotion regulation strategies
Source: Commun Psychol. 2025 Nov 28;3:180. doi: 10.1038/s44271-025-00368-6 (PMC12673100; doi:10.1038/s44271-025-00368-6)
Supplement: Supplementary file 2 — Supplement [file 44271_2025_368_MOESM2_ESM.pdf]

Supplementary Information for: Laughter Regulation in Solitary and Social Contexts: Differential Effects of Suppression, Reappraisal, and Distraction

Vanessa Mitschke, Annika Ziereis, Sriranjani Manivasagam, Anne Schacht

16 Oktober, 2025

Contents

Instructions for the emotion regulation tasks (Experient 1 and 2) . . . 2
Experiment 1 . . . 2
Positive and Negative Affect Scales (PANAS) . . . 2
Model 1: Ratings of funniness . . . 3
Model 2: Changes in muscle activity due to emotion regulation strategies . . . 4
Model 2B: Changes in CS activity due to emotion regulation strategies . . . 5
Model 3: Muscle activity and subjective funniness . . . 6
Model 3B. Ratings of funniness . . . 7
Experiment 2 . . . 8
Positive and Negative Affect Scales (PANAS) . . . 8
Model 1: Ratings of funniness . . . 9
Model 2: Changes in muscle activity due to emotion regulation strategies . . . 10
Model 2B: Changes in CS activity due to emotion regulation strategies . . . 11
Model 3: Muscle activity and subjective funniness . . . 12
Model 3B. Ratings of funniness . . . 13
Experiment 1 & 2 pooled . . . 14
Model 1: Ratings of funniness . . . 14
Model 3B. Ratings of funniness . . . 15
Experiment 3 . . . 16
Positive and Negative Affect Scales (PANAS) . . . 16
Model 1: Ratings of funniness . . . 17
Model 2: Muscle Inhibition modulated by Social Feedback . . . 18
Model 3: Influence of Humorous Stimuli on Mimicry Suppression . . . 19

Correspondence: Goßlerstr. 14, 37073 Goettingen, Germany Email: vanessa.mitschke@uni-goettingen.de

Instructions for the emotion regulation tasks (Experient 1 and 2)

Reappraisal Instruction (translated from German): *"During the next 35 recordings, we would like to ask you to actively re-evaluate the recordings. To do this, you should mentally change the meaning of the recordings. For example, you could imagine that you are just an uninvolved listener who is simply listening to the questions and trying to understand their meaning. You can also change the meaning of the recordings by focusing on possible negative aspects of the audio clips. In any case, it is important that you listen attentively and do not think about anything else other than the recordings while doing so."*

Expressive Suppression Instruction (translated from German): *"During the next 35 recordings, we would like to ask you not to show how you feel about the recordings while you are listening to them. From the outside, no one should see what you are experiencing while listening to the audio clips (sensations, thoughts, feelings). To do this, try to relax your facial muscles so that someone observing you cannot guess what you are experiencing (like putting on a poker face while playing cards). It is important that you do not think about anything else, tune out, or change the situation in your thoughts while doing this."*

Distraction Instruction (translated from German): *"During the next 20 jokes, we'd like to ask you to search for Wally in the hidden object poster in front of you. You should still listen to the jokes, but your focus should be on finding Wally. If you find Wally, please continue searching for Sam. After each joke, we'll ask you to rate the joke."*

$instructions included images of the characters$

Experiment 1

Positive and Negative Affect Scales (PANAS)

Table S1:
Experiment 1 PANAS: Repeated measures ANOVA, Time (pre vs. post) × Valence (positive vs. negative)

	df_{num}	df_{den}	MSE	F	p	η^2_G	η^2_P
valence	1	39	0.40	319.83	<.001	0.75	0.89
time	1	39	0.08	38.10	<.001	0.07	0.49
valence:time	1	39	0.13	9.98	.003	0.03	0.20

Table S2:
Experiment 1 PANAS: Estimated Marginal Means - Time × Valence

Valence	Time	Mean	SE	df	CI_l	CI_u
Positive	pre	3.24	0.09	39	3.06	3.42
	post	2.78	0.12	39	2.53	3.02
Negative	pre	1.28	0.04	39	1.19	1.36
	post	1.17	0.05	39	1.08	1.27

Model 1: Ratings of funniness

Formula: humor_rating ~ condition + (1 + condition |subject) + (1|JokeID)

Information on the data included in the model:

- Jokes only

Table S3:
Results (Experiment 1): Humor rating as a function of condition

	β	SE	t	CI_l	CI_u	$Stab_{min}$	$Stab_{max}$	p_{Satter}	$R^2_{partial}$	CI_{lR2}	CI_{uR2}	LRT:Model	χ^2	df	p
(Intercept)	2.29	0.10	23.59	2.10	2.48	2.26	2.32	.000	-	-	-	(Intercept)	-	-	-
condition_reappraisal	-0.21	0.09	-2.30	-0.39	-0.04	-0.25	-0.18	.027	.0052	.0015	.0110	condition	5.39	2	.068
condition_suppression	-0.04	0.06	-0.57	-0.16	0.08	-0.05	-0.01	.574	.0001	.0000	.0021				

Notes: β = model estimate, SE = standard error of the estimate, CI = lower and upper 95% bootstrapped confidence intervals, Stab = estimate ranges leaving out one participant at a time, p_{Satter} = Satterwhite approximated p-values, $R^2_{partial}$ = partial R^2 with lower and upper confidence intervals, LRT = Likelihood ratio test. The model's overall $R^2_{partial}$ was: .0059 [.0022,.0124]

Figure S1:
Model-predicted humor rating by condition.

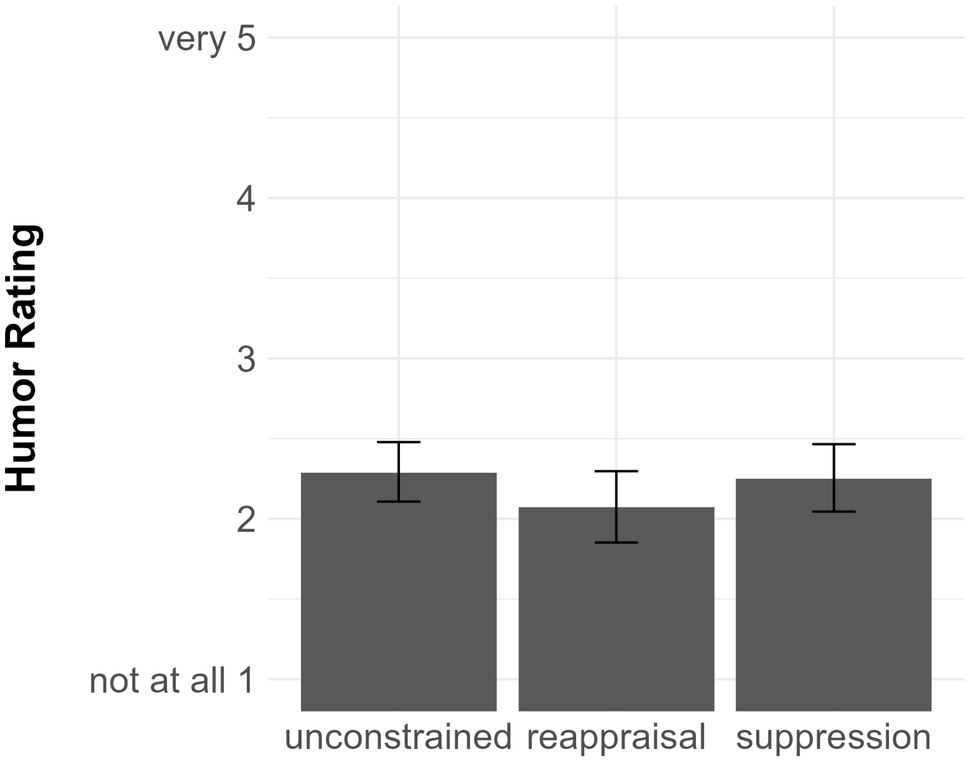

Notes: The error bars show the 95% bootstrapped CI of the model predictions

Model 2: Changes in muscle activity due to emotion regulation strategies

Formula: Index ~ condition * segment + (1 + condition * segment|ID) + (1 + condition * segment|jokeID)

Information on the data included in the model:

- single trials of all conditions (unconstrained, suppression, reappraisal)
- the reference level for the factor condition was "unconstrained"
- time windows (segments) were z-transformed for model stability
- Index was calculated based on the within-subject z-transformed muscle.

Table S4:
Results of Model 2 (Experiment 1): Smile index as a function of condition over time

	β	SE	t	CI_l	CI_u	$Stab_{min}$	$Stab_{max}$	p_{Satter}	$R^2_{partial}$	CI_{lR2}	CI_{uR2}	LRT:Model	χ^2	df	p
(Intercept)	-1.09	0.08	-13.81	-1.25	-0.93	-1.13	-1.07	.000	-	-	-	(Intercept)	-	-	-
conditionreappraisal	0.16	0.07	2.30	0.02	0.30	0.14	0.19	.028	.0003	.0000	.0008				
conditionsuppression	0.25	0.06	4.34	0.14	0.36	0.24	0.28	.000	.0007	.0003	.0015	condition	24.7	2	<.001
segment	0.23	0.02	11.05	0.18	0.27	0.22	0.23	.000	.0461	.0416	.0508	segment	53.97	1	<.001
conditionreappraisal:segment	-0.11	0.02	-5.35	-0.15	-0.07	-0.11	-0.10	.000	.0053	.0038	.0071				
conditionsuppression:segment	-0.17	0.02	-8.48	-0.21	-0.13	-0.18	-0.16	.000	.0139	.0114	.0167	condition:segment	48.89	2	<.001

Notes: β = model estimate, SE = standard error of the estimate, CI = lower and upper 95% bootstrapped confidence intervals, Stab = estimate ranges leaving out one participant at a time, p_{Satter} = Satterwhite approximated p-values, $R^2_{partial}$ = partial R^2 with lower and upper confidence intervals, LRT = Likelihood ratio test. The model's overall $R^2_{partial}$ was: .0965 [.0905,.1030].Each muscle (ZM, OO, CS) z-transformed to a mean of 0 and sd of 1 for every subject to calculate Smile Index.

Figure S2:
Model-predicted Smile Index by condition over time.

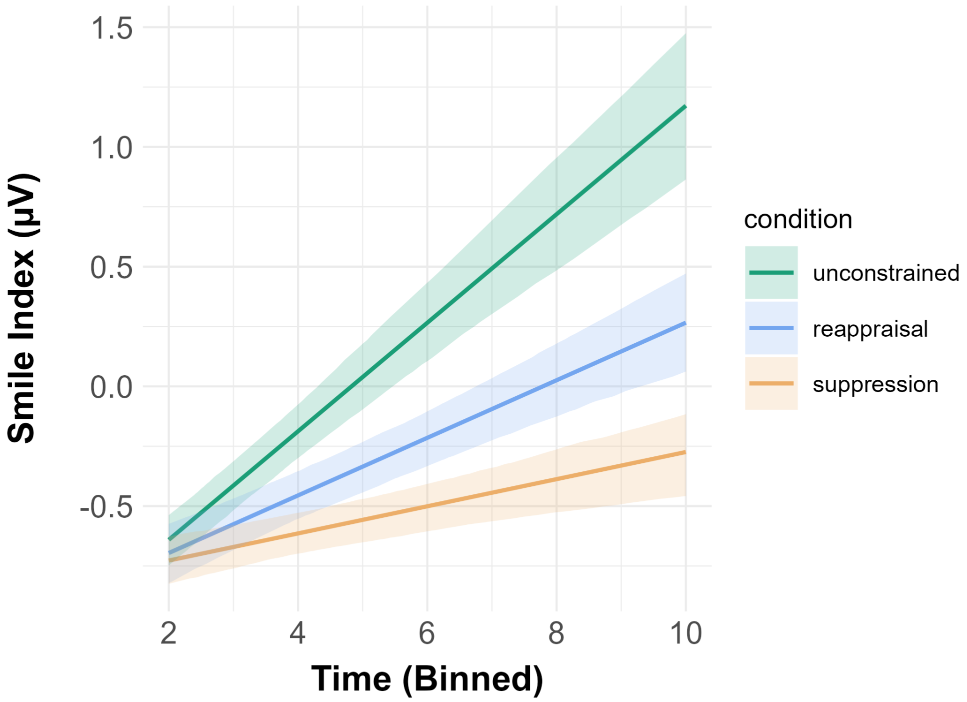

Notes: The shaded areas show the 95% bootstrapped CI of the model predictions

Model 2B: Changes in CS activity due to emotion regulation strategies

Formula: CS ~ condition * segment + (1 + condition * segment|ID) + (1 + condition * segment|jokeID)

Information on the data included in the model:

- single trials of all conditions (unconstrained, suppression, reappraisal)
- the reference level for the factor condition was "unconstrained"
- time windows (segments) were z-transformed for model stability
- CS activity refers to the within-subject z-transformed muscle.

Table S5:
Results of Model 2B (Experiment 1): CS activity as a function of condition over time

	β	SE	t	CI_l	CI_u	$Stab_{min}$	$Stab_{max}$	p_{Satter}	$R^2_{partial}$	CI_{lR2}	CI_{uR2}	LRT:Model	χ^2	df	p
(Intercept)	0.01	0.04	0.22	-0.07	0.09	0.00	0.02	.829	-	-	-	(Intercept)	-	-	-
conditionreappraisal	-0.09	0.06	-1.48	-0.21	0.03	-0.11	-0.07	.149	.0023	.0013	.0035	condition	5.05	2	.080
conditionsuppression	0.01	0.06	0.13	-0.12	0.12	-0.01	0.02	.900	.0000	.0000	.0002				
segment	-0.03	0.02	-1.48	-0.07	0.01	-0.04	-0.02	.151	.0005	.0001	.0011	segment	11.41	1	<.001
conditionreappraisal:segment	-0.05	0.02	-2.12	-0.09	-0.01	-0.06	-0.04	.041	.0007	.0002	.0014	condition:segment	4.36	2	.113
conditionsuppression:segment	-0.02	0.02	-1.20	-0.06	0.02	-0.03	-0.01	.241	.0002	.0000	.0006				

Notes: β = model estimate, SE = standard error of the estimate, CI = lower and upper 95% bootstrapped confidence intervals, Stab = estimate ranges leaving out one participant at a time, p_{Satter} = Satterwhite approximated p-values, $R^2_{partial}$ = partial R^2 with lower and upper confidence intervals, LRT = Likelihood ratio test. The model's overall $R^2_{partial}$ was: .0089 [.0070,.0112].CS was z-transformed to a mean of 0 and sd of 1 for every subject.

Figure S3:
Model-predicted CS activity by condition over time.

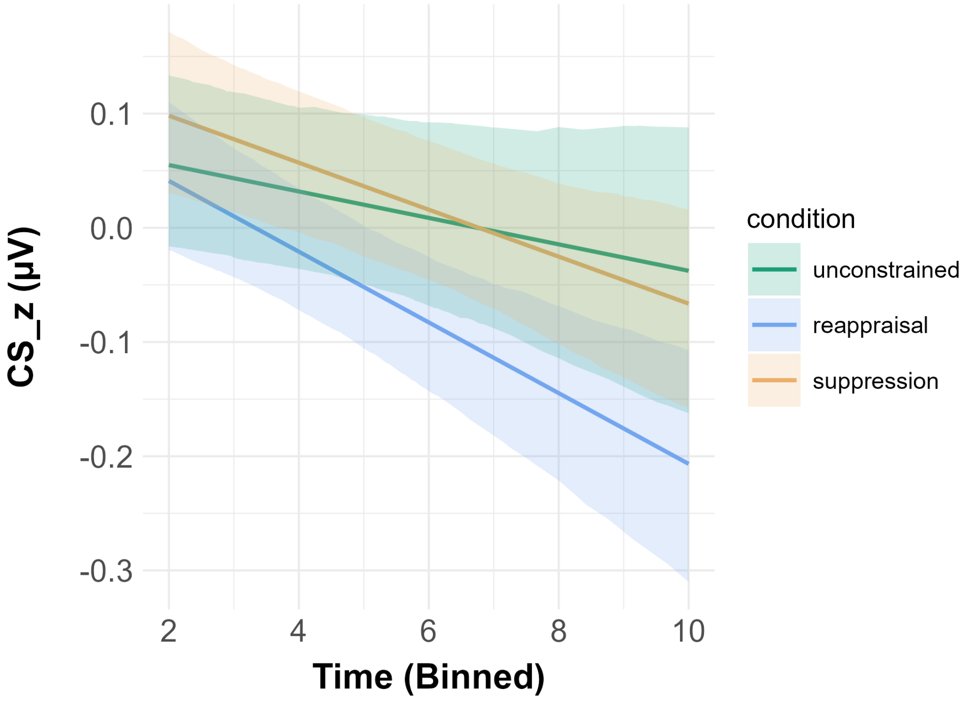

Notes: The shaded areas show the 95% bootstrapped CI of the model predictions

Model 3: Muscle activity and subjective funniness

Formula: Smile Index ~ humrating * segment + (1 + humrating + segment|ID) + (1 + humrating + segment|jokeID)

Information on the data included in the model:

- single trials of the unconstrained condition only
- the reference level for the humor rating of the jokes (factor) was 1 (not funny)
- trial-based smile index and time windows (segments) were z-transformed for model stability
- Index was calculated based on the within-subject z-transformed muscle.

Table S6:

Results of Model 3 (Experiment 1): Smile index as a function of funniness ratings over time

	β	SE	t	CI_l	CI_u	$Stab_{min}$	$Stab_{max}$	p_{Satter}	$R^2_{partial}$	CI_{lR2}	CI_{uR2}	LRT:Model	χ^2	df	p
(Intercept)	-0.46	0.10	-4.87	-0.67	-0.28	-0.50	-0.43	.000	-	-	-	(Intercept)	-	-	-
humrating2	0.50	0.13	3.73	0.25	0.75	0.47	0.56	.000	.0122	.0082	.0169				
humrating3	1.19	0.18	6.55	0.83	1.54	1.15	1.26	.000	.0541	.0456	.0631				
humrating4	2.16	0.25	8.76	1.67	2.67	2.06	2.24	.000	.1184	.1068	.1305				
humrating5	2.32	0.21	10.98	1.80	2.80	1.95	2.57	.000	.0355	.0286	.0431	humrating	58.98	4	<.001
segment	0.16	0.05	3.61	0.07	0.25	0.15	0.18	.001	.0029	.0011	.0054	segment	41.03	1	<.001
humrating2:segment	0.27	0.07	4.16	0.15	0.40	0.26	0.29	.000	.0035	.0016	.0063				
humrating3:segment	0.74	0.09	8.04	0.55	0.92	0.71	0.76	.000	.0216	.0162	.0277				
humrating4:segment	1.42	0.14	9.92	1.13	1.71	1.36	1.48	.000	.0557	.0472	.0649				
humrating5:segment	1.47	0.16	9.09	1.08	1.84	1.33	1.73	.000	.0153	.0108	.0206	humrating:segment	69.44	4	<.001

Notes: β = model estimate, SE = standard error of the estimate, CI = lower and upper 95% bootstrapped confidence intervals, Stab = estimate ranges leaving out one participant at a time, p_{Satter} = Satterwhite approximated p-values, $R^2_{partial}$ = partial R^2 with lower and upper confidence intervals, LRT = Likelihood ratio test. The model's overall $R^2_{partial}$ was: .2592 [.2456,.2739].Each muscle (ZM, OO, CS) z-transformed to a mean of 0 and sd of 1 for every subject to calculate Smile Index.

Figure S4:

Model-predicted Smile Index by humor ratings over time.

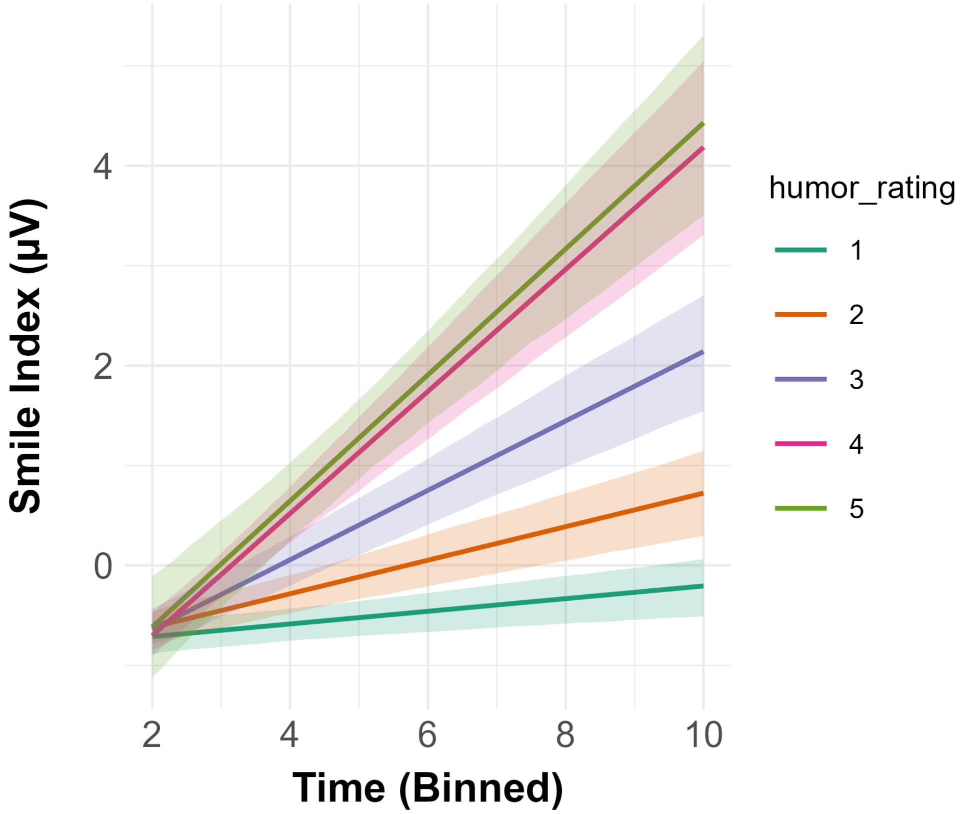

Notes: The shaded areas show the 95% bootstrapped CI of the model predictions

Model 3B. Ratings of funniness

Formula: humrating ~ condition * Index + (1 + condition * Index |ID) + (1 + condition * Index |jokeID)

Information on the data included in the model:

- single trials of all conditions
- the reference level for the factor condition was "unconstrained"
- trial-based smile index was z-transformed for model stability
- Index was calculated based on the within-subject z-transformed muscle.

Table S7:
Results of Model 3B (Experiment 1): Joke ratings as a function of condition and muscle activation

	β	SE	t	CI_l	CI_u	$Stab_{min}$	$Stab_{max}$	p_{Satter}	$R^2_{partial}$	CI_{lR2}	CI_{uR2}	LRT:Model	χ^2	df	p
(Intercept)	2.19	0.09	23.17	2.00	2.38	2.16	2.22	.000	-	-	-	(Intercept)	-	-	-
conditionreappraisal	-0.07	0.10	-0.72	-0.25	0.10	-0.11	-0.03	.482	.0006	.0002	.0012				
conditionsuppression	0.23	0.08	2.80	0.07	0.38	0.20	0.25	.007	.0056	.0040	.0074	condition	10.76	2	.005
Index	0.15	0.02	8.23	0.11	0.19	0.15	0.16	.000	.0204	.0173	.0237	Index	51.38	1	<.001
conditionreappraisal:Index	0.02	0.03	0.91	-0.03	0.08	0.02	0.03	.371	.0002	.0000	.0007				
conditionsuppression:Index	0.12	0.04	2.75	0.03	0.20	0.10	0.13	.009	.0031	.0020	.0045	condition:Index	7.32	2	.026

Notes: β = model estimate, SE = standard error of the estimate, CI = lower and upper 95% bootstrapped confidence intervals, Stab = estimate ranges leaving out one participant at a time, p_{Satter} = Satterwhite approximated p-values, $R^2_{partial}$ = partial R^2 with lower and upper confidence intervals, LRT = Likelihood ratio test. The model's overall $R^2_{partial}$ was: .0620 [.0570,.0675].Each muscle (ZM, OO, CS) z-transformed to a mean of 0 and sd of 1 for every subject to calculate Smile Index.

Figure S5:
Model-predicted humor ratings by condition and muscle activation.

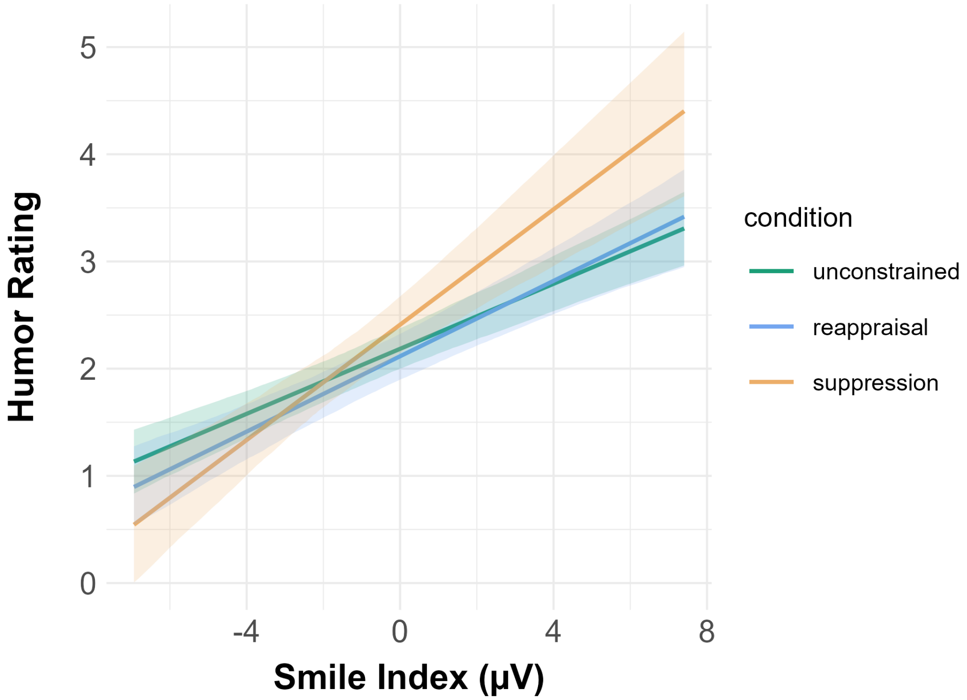

Notes: The shaded areas show the 95% bootstrapped CI of the model predictions

Experiment 2

Positive and Negative Affect Scales (PANAS)

Table S8:
Experiment 2 PANAS: Repeated measures ANOVA, Time (pre vs. post) × Valence (positive vs. negative)

	df_{num}	df_{den}	MSE	F	p	η^2_G	η^2_P
valence	1	41	0.44	287.70	<.001	0.77	0.88
time	1	41	0.05	17.15	<.001	0.02	0.29
valence:time	1	41	0.05	0.22	.638	0.00	0.01

Table S9:
Experiment 2 PANAS: Estimated Marginal Means - Time × Valence

Valence	Time	Mean	SE	df	CI_l	CI_u
Negative	post	1.14	0.03	41	1.08	1.19
	pre	1.26	0.04	41	1.17	1.34
Positive	post	2.86	0.11	41	2.64	3.08
	pre	3.02	0.09	41	2.83	3.20

Model 1: Ratings of funniness

Formula: humor_rating ~ condition + (1 + condition |subject) + (1|JokeID)

Information on the data included in the model:

- Jokes only

Table S10:
Results (Experiment 2): Humor rating as a function of condition

	β	SE	t	CI_l	CI_u	$Stab_{min}$	$Stab_{max}$	p_{Satter}	$R^2_{partial}$	CI_{lR2}	CI_{uR2}	LRT:Model	χ^2	df	p
(Intercept)	2.01	0.08	25.95	1.86	2.17	1.98	2.03	.000	-	-	-	(Intercept)	-	-	-
condition_disengagement	-0.40	0.06	-6.27	-0.53	-0.28	-0.43	-0.38	.000	.0211	.0124	.0321				
condition_reappraisal	-0.30	0.08	-3.74	-0.46	-0.15	-0.33	-0.27	.001	.0114	.0052	.0199	condition	30.29	3	<.001
condition_suppression	-0.11	0.06	-1.84	-0.23	0.02	-0.15	-0.09	.071	.0017	.0001	.0058				

Notes: β = model estimate, SE = standard error of the estimate, CI = lower and upper 95% bootstrapped confidence intervals, Stab = estimate ranges leaving out one participant at a time, p_{Satter} = Satterwhite approximated p-values, $R^2_{partial}$ = partial R^2 with lower and upper confidence intervals, LRT = Likelihood ratio test. The model's overall $R^2_{partial}$ was: .0253 [.0162,.0378]

Figure S6:
Model-predicted humor rating by condition.

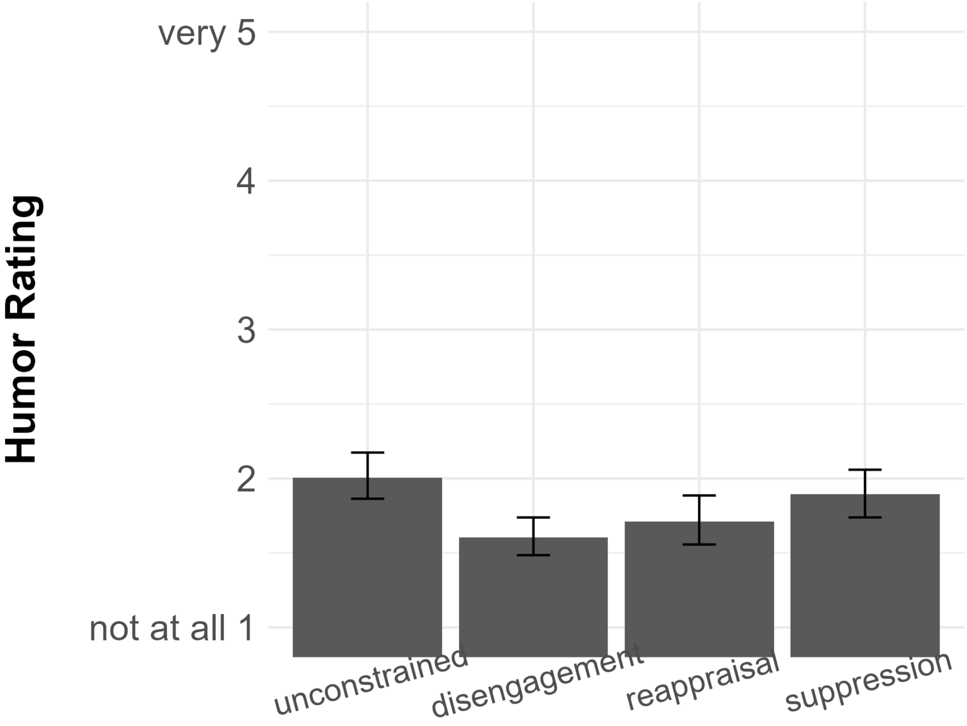

Notes: The error bars show the 95% bootstrapped CI of the model predictions

Model 2: Changes in muscle activity due to emotion regulation strategies

Formula: Index ~ condition * segment + (1 + condition * segment|ID) + (1 + condition * segment|jokeID)

Information on the data included in the model:

- single trials of all conditions (unconstrained, suppression, reappraisal, distraction)
- the reference level for the factor condition was "unconstrained"
- time windows (segments) were z-transformed for model stability
- Index was calculated based on the within-subject z-transformed muscle.

Table S11:

Results of Model 2 (Experiment 1): Smile index as a function of condition over time

	β	SE	t	CI_l	CI_u	$Stab_{min}$	$Stab_{max}$	p_{Satter}	$R^2_{partial}$	CI_{lR2}	CI_{uR2}	LRT:Model	χ^2	df	p
(Intercept)	0.17	0.08	2.11	0.02	0.33	0.14	0.20	.040	-	-	-	(Intercept)	-	-	-
conditiondistraction	-0.54	0.12	-4.68	-0.77	-0.32	-0.57	-0.49	.000	.0185	.0157	.0216				
conditionreappraisal	-0.43	0.11	-4.07	-0.66	-0.23	-0.46	-0.39	.000	.0119	.0096	.0144				
conditionsuppression	-0.54	0.09	-5.73	-0.73	-0.37	-0.57	-0.50	.000	.0189	.0160	.0220	condition	25.99	3	<.001
segment	0.55	0.06	8.63	0.43	0.68	0.53	0.57	.000	.0370	.0330	.0412	segment	47.53	1	<.001
conditiondistraction:segment	-0.36	0.07	-4.99	-0.52	-0.23	-0.39	-0.33	.000	.0086	.0067	.0108				
conditionreappraisal:segment	-0.34	0.07	-4.90	-0.48	-0.21	-0.36	-0.31	.000	.0073	.0055	.0093				
conditionsuppression:segment	-0.45	0.06	-7.30	-0.58	-0.33	-0.47	-0.42	.000	.0130	.0106	.0157	condition:segment	37.49	3	<.001

Notes: β = model estimate, SE = standard error of the estimate, CI = lower and upper 95% bootstrapped confidence intervals, Stab = estimate ranges leaving out one participant at a time, p_{Satter} = Satterwhite approximated p-values, $R^2_{partial}$ = partial R^2 with lower and upper confidence intervals, LRT = Likelihood ratio test. The model's overall $R^2_{partial}$ was: .0689 [.0638,.0746].Each muscle (ZM, OO, CS) z-transformed to a mean of 0 and sd of 1 for every subject to calculate Smile Index.

Figure S7:

Model-predicted Smile Index by condition over time.

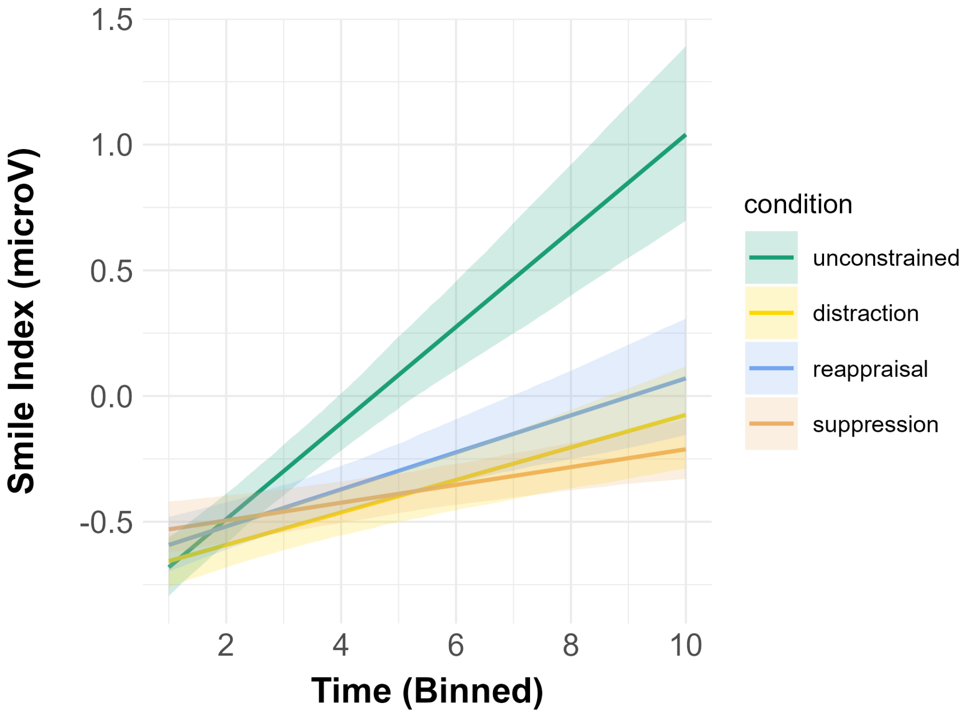

Notes: The shaded areas show the 95% bootstrapped CI of the model predictions

Model 2B: Changes in CS activity due to emotion regulation strategies

Formula: CS ~ condition * segment + (1 + condition * segment|ID) + (1 + condition * segment|jokeID)

Information on the data included in the model:

- single trials of all conditions (unconstrained, suppression, reappraisal, distraction)
- the reference level for the factor condition was "unconstrained"
- time windows (segments) were z-transformed for model stability
- CS activity refers to the within-subject z-transformed muscle.

Table S12:
Results of Model 2B (Experiment 2): CS activity as a function of condition over time

	β	SE	t	CI_l	CI_u	$Stab_{min}$	$Stab_{max}$	p_{Satter}	$R^2_{partial}$	CI_{lR2}	CI_{uR2}	LRT:Model	χ^2	df	p
(Intercept)	0.00	0.04	0.07	-0.07	0.07	-0.01	0.02	.944	-	-	-	(Intercept)	-	-	-
conditiondistraction	0.01	0.06	0.11	-0.12	0.13	-0.02	0.03	.910	.0000	.0000	.0002				
conditionreappraisal	-0.01	0.06	-0.22	-0.12	0.09	-0.03	0.01	.831	.0000	.0000	.0003	condition	0.15	3	.985
conditionsuppression	0.00	0.05	-0.02	-0.09	0.09	-0.02	0.02	.988	.0000	.0000	.0002				
segment	-0.04	0.03	-1.51	-0.10	0.01	-0.05	-0.03	.141	.0008	.0003	.0015	segment	25.42	1	<.001
conditiondistraction:segment	-0.08	0.04	-1.91	-0.15	0.01	-0.09	-0.06	.065	.0013	.0006	.0022				
conditionreappraisal:segment	-0.04	0.03	-1.15	-0.10	0.02	-0.05	-0.03	.259	.0003	.0000	.0008	condition:segment	6.24	3	.101
conditionsuppression:segment	-0.01	0.03	-0.42	-0.07	0.04	-0.02	0.00	.682	.0000	.0000	.0003				

Notes: β = model estimate, SE = standard error of the estimate, CI = lower and upper 95% bootstrapped confidence intervals, Stab = estimate ranges leaving out one participant at a time, p_{Satter} = Satterwhite approximated p-values, $R^2_{partial}$ = partial R^2 with lower and upper confidence intervals, LRT = Likelihood ratio test. The model's overall $R^2_{partial}$ was: .0089 [.0070,.0112].CS was z-transformed to a mean of 0 and sd of 1 for every subject.

Figure S8:
Model-predicted Corrugator activity by condition over time.

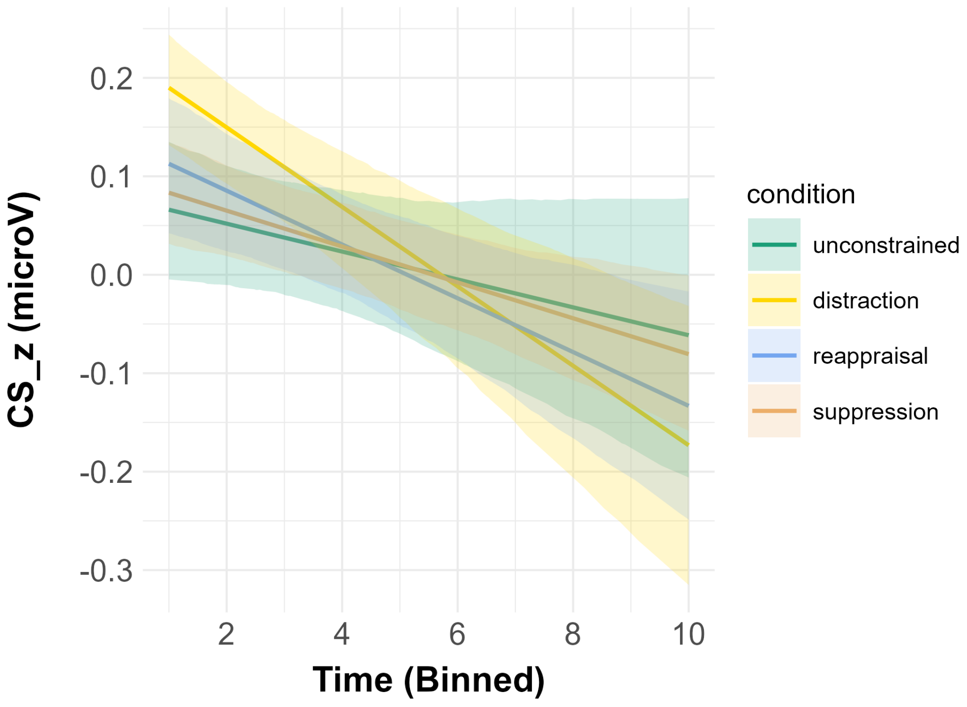

Notes: The shaded areas show the 95% bootstrapped CI of the model predictions

Model 3: Muscle activity and subjective funniness

Formula: Smile Index ~ humrating * segment + (1 + humrating + segment|ID) + (1 + humrating + segment|jokeID)

Information on the data included in the model:

- single trials of the unconstrained condition only
- the reference level for the humor rating of the jokes (factor) was 1 (not funny)
- trial-based smile index and time windows (segments) were z-transformed for model stability
- Index was calculated based on the within-subject z-transformed muscle.

Table S13:

Results of Model 3 (Experiment 2): Smile index as a function of funniness ratings over time

	β	SE	t	CI_l	CI_u	$Stab_{min}$	$Stab_{max}$	p_{Satter}	$R^2_{partial}$	CI_{lR2}	CI_{uR2}	LRT:Model	χ^2	df	p
(Intercept)	-0.34	0.10	-3.39	-0.53	-0.14	-0.38	-0.30	.001	-	-	-	(Intercept)	-	-	-
humrating2	0.58	0.16	3.65	0.27	0.90	0.53	0.63	.001	.0190	.0133	.0256				
humrating3	1.41	0.25	5.62	0.94	1.88	1.35	1.48	.000	.0699	.0592	.0813				
humrating4	1.65	0.26	6.47	1.12	2.18	1.53	1.75	.000	.0528	.0434	.0630				
humrating5	2.74	0.44	6.26	1.60	4.03	2.25	3.29	.000	.0163	.0111	.0226	humrating	37.43	4	<.001
segment	0.22	0.07	3.06	0.08	0.37	0.19	0.25	.004	.0066	.0034	.0108	segment	21.98	1	<.001
humrating2:segment	0.32	0.10	3.34	0.14	0.52	0.28	0.35	.002	.0059	.0029	.0099				
humrating3:segment	1.00	0.16	6.13	0.70	1.34	0.95	1.06	.000	.0371	.0291	.0459				
humrating4:segment	1.31	0.19	6.96	0.94	1.70	1.20	1.38	.000	.0347	.0270	.0433				
humrating5:segment	2.56	0.38	6.78	1.61	3.64	2.23	3.05	.000	.0140	.0091	.0198	humrating:segment	42.02	4	<.001

Notes: β = model estimate, SE = standard error of the estimate, CI = lower and upper 95% bootstrapped confidence intervals, Stab = estimate ranges leaving out one participant at a time, p_{Satter} = Satterwhite approximated p-values, $R^2_{partial}$ = partial R^2 with lower and upper confidence intervals, LRT = Likelihood ratio test. The model's overall $R^2_{partial}$ was: .2237 [.2087,.2403].Each muscle (ZM, OO, CS) z-transformed to a mean of 0 and sd of 1 for every subject to calculate Smile Index.

Figure S9:

Model-predicted Smile Index by humor ratings over time.

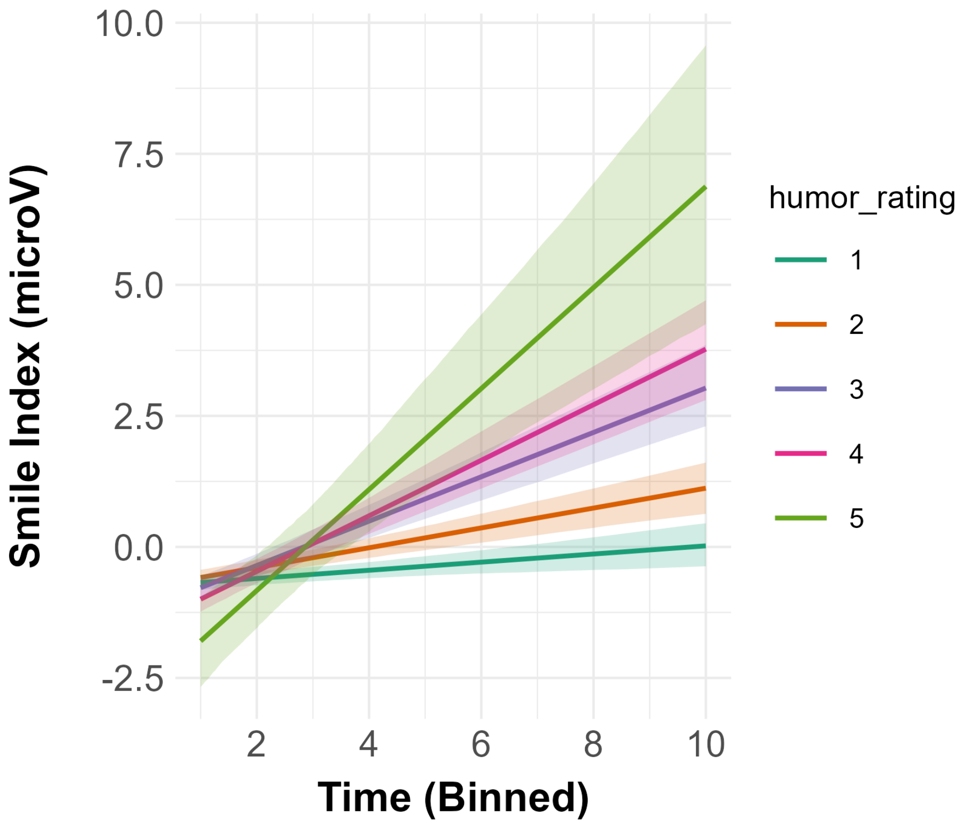

Notes: The shaded areas show the 95% bootstrapped CI of the model predictions

Model 3B. Ratings of funniness

Formula: humrating ~ condition * Index + (1 + condition * Index |ID) + (1 + condition * Index |jokeID)

Information on the data included in the model:

- single trials of all conditions
- the reference level for the factor condition was "unconstrained"
- trial-based smile index was z-transformed for model stability
- Index was calculated based on the within-subject z-transformed muscle.

Table S14:

Results of Model 3B (Experiment 2): Joke ratings as a function of condition and muscle activation

	β	SE	t	CI_l	CI_u	$Stab_{min}$	$Stab_{max}$	p_{Satter}	$R^2_{partial}$	CI_{lR2}	CI_{uR2}	LRT:Model	χ^2	df	p
(Intercept)	1.90	0.08	23.89	1.76	2.08	1.88	1.93	.000	-	-	-	(Intercept)	-	-	-
conditiondistraction	-0.28	0.07	-4.03	-0.42	-0.15	-0.32	-0.25	.000	.0104	.0083	.0128				
conditionreappraisal	-0.19	0.09	-2.18	-0.37	-0.02	-0.23	-0.17	.034	.0047	.0033	.0064				
conditionsuppression	0.10	0.08	1.25	-0.06	0.27	0.06	0.13	.219	.0013	.0006	.0023	condition	23.3	3	<.001
Index	0.10	0.02	5.48	0.06	0.13	0.09	0.10	.000	.0090	.0070	.0112	Index	35.53	1	<.001
conditiondistraction:Index	-0.03	0.03	-1.22	-0.08	0.02	-0.04	-0.02	.230	.0003	.0001	.0009				
conditionreappraisal:Index	0.00	0.03	0.11	-0.06	0.06	-0.01	0.01	.910	.0000	.0000	.0002				
conditionsuppression:Index	0.15	0.06	2.77	0.05	0.27	0.13	0.18	.009	.0044	.0031	.0060	condition:Index	9.26	3	.026

Notes: β = model estimate, SE = standard error of the estimate, CI = lower and upper 95% bootstrapped confidence intervals, Stab = estimate ranges leaving out one participant at a time, p_{Satter} = Satterwhite approximated p-values, $R^2_{partial}$ = partial R^2 with lower and upper confidence intervals, LRT = Likelihood ratio test. The model's overall $R^2_{partial}$ was: .0508 [.0463,.0558].Each muscle (ZM, OO, CS) z-transformed to a mean of 0 and sd of 1 for every subject to calculate Smile Index.

Figure S10:

Model-predicted humor ratings by condition and muscle activation.

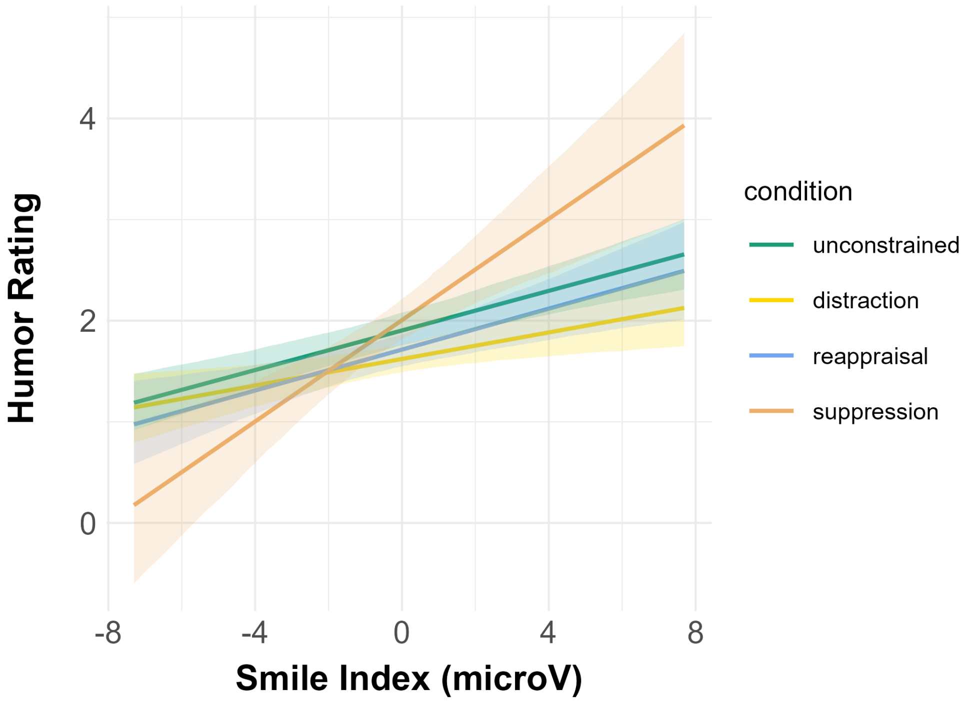

Notes: The shaded areas show the 95% bootstrapped CI of the model predictions

Experiment 1 & 2 pooled

Model 1: Ratings of funniness

Formula: humor_rating ~ condition + (1 + condition | subject) + (1 | JokeID) + (1 + condition | study)

Information on the data included in the model:

- Jokes only
- Conditions only present in both studies (reappraisal, unconstrained, suppression)

Table S15:
Results (Experiments 1&2): Humor rating as a function of condition

	β	SE	t	CI_l	CI_u	$Stab_{min}$	$Stab_{max}$	p_{Satter}	$R^2_{partial}$	CI_{lR2}	CI_{uR2}	LRT:Model	χ^2	df	p
(Intercept)	2.12	0.13	16.21	1.85	2.37	2.10	2.13	.002	-	-	-	(Intercept)	-	-	-
condition_reappraisal	-0.26	0.07	-3.76	-0.39	-0.12	-0.27	-0.24	.012	.0082	.0042	.0134	condition	5.27	2	.072
condition_suppression	-0.07	0.05	-1.41	-0.18	0.03	-0.09	-0.06	.240	.0007	.0000	.0027				

Notes: β = model estimate, SE = standard error of the estimate, CI = lower and upper 95% bootstrapped confidence intervals, Stab = estimate ranges leaving out one participant at a time, p_{Satter} = Satterwhite approximated p-values, $R^2_{partial}$ = partial R^2 with lower and upper confidence intervals, LRT = Likelihood ratio test. The model's overall $R^2_{partial}$ was: .0086 [.0047,.0141]

Figure S11:
Model-predicted humor rating by condition.

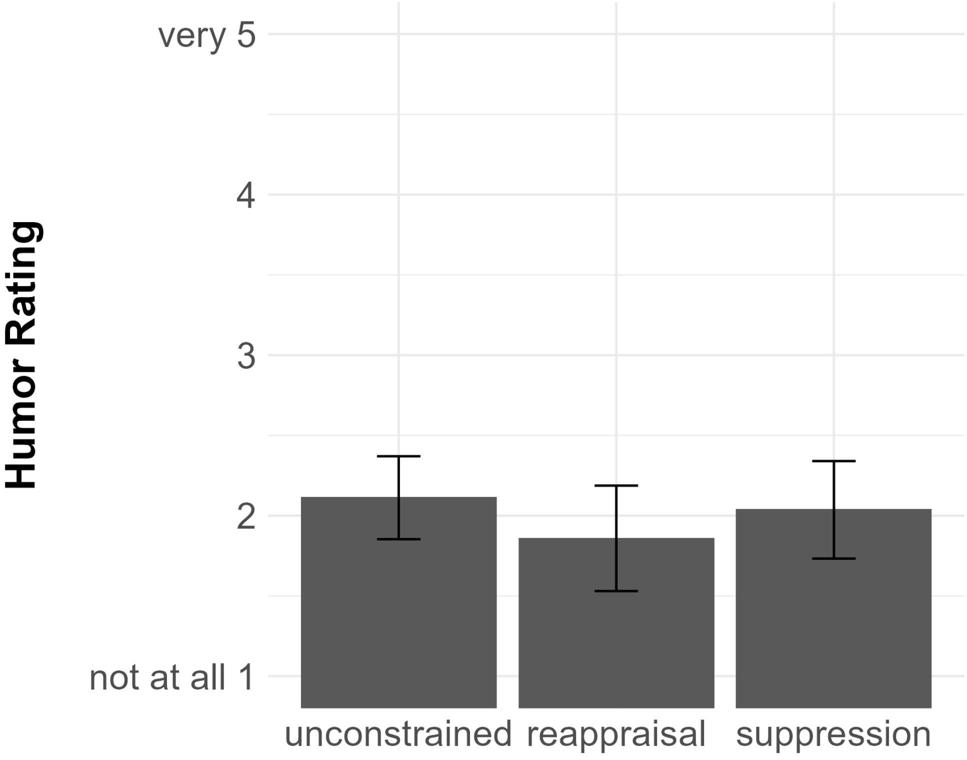

Notes: The error bars show the 95% bootstrapped CI of the model predictions

Model 3B. Ratings of funniness

Formula: humrating ~ condition * Index + (1 + condition * Index |ID) + (1 + condition * Index |jokeID)

Information on the data included in the model:

- single trials of all conditions
- the reference level for the factor condition was "unconstrained"
- trial-based smile index was z-transformed for model stability
- Index was calculated based on the within-subject z-transformed muscle.

Table S16:
Results of Model 3B (Experiments 1&2): Joke ratings as a function of condition and muscle activation

	β	SE	t	CI_l	CI_u	$Stab_{min}$	$Stab_{max}$	p_{Satter}	$R^2_{partial}$	CI_{lR2}	CI_{uR2}	LRT:Model	χ^2	df	p
(Intercept)	2.04	0.07	30.24	1.90	2.17	2.03	2.06	.000	-	-	-	(Intercept)	-	-	-
conditionreappraisal	-0.13	0.07	-1.93	-0.26	0.00	-0.15	-0.11	.057	.0021	.0014	.0030				
conditionsuppression	0.14	0.06	2.44	0.03	0.26	0.13	0.16	.016	.0026	.0018	.0035	condition	13.38	2	.001
Index	0.13	0.01	10.23	0.11	0.16	0.13	0.14	.000	.0167	.0146	.0189	Index	94.45	1	<.001
conditionreappraisal:Index	0.02	0.02	1.07	-0.02	0.07	0.02	0.03	.290	.0002	.0000	.0005				
conditionsuppression:Index	0.13	0.03	4.15	0.06	0.18	0.12	0.13	.000	.0037	.0027	.0048	condition:Index	16.83	2	<.001

Notes: β = model estimate, SE = standard error of the estimate, CI = lower and upper 95% bootstrapped confidence intervals, Stab = estimate ranges leaving out one participant at a time, p_{Satter} = Satterwhite approximated p-values, $R^2_{partial}$ = partial R^2 with lower and upper confidence intervals, LRT = Likelihood ratio test. The model's overall $R^2_{partial}$ was: .0545 [.0509,.0583].Each muscle (ZM, OO, CS) z-transformed to a mean of 0 and sd of 1 for every subject to calculate Smile Index.

Figure S12:
Model-predicted humor ratings by condition and muscle activation.

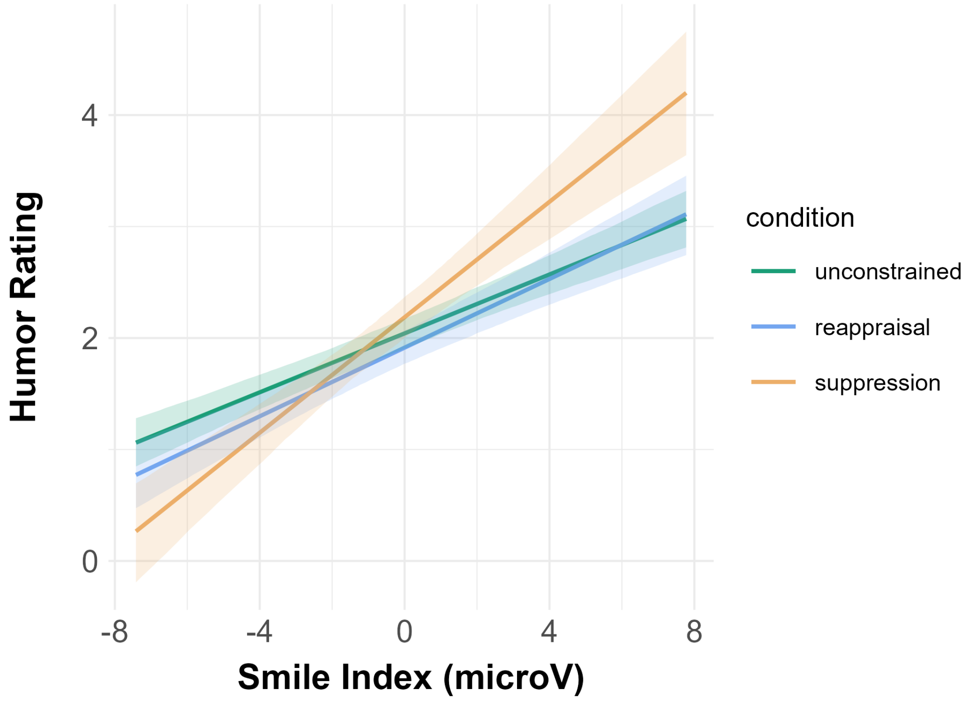

Notes: The shaded areas show the 95% bootstrapped CI of the model predictions

Experiment 3

Positive and Negative Affect Scales (PANAS)

Table S17:
Experiment 3 PANAS: Repeated measures ANOVA, Time (pre vs. post) × Valence (positive vs. negative)

	df_{num}	df_{den}	MSE	F	p	η^2_G	η^2_P
valence	1	40	0.40	343.86	<.001	0.81	0.90
time	1	40	0.03	38.98	<.001	0.04	0.49
valence:time	1	40	0.08	2.80	.102	0.01	0.07

Table S18:
Experiment 3 PANAS: Estimated Marginal Means - Time × Valence

Valence	Time	Mean	SE	df	CI_l	CI_u
Positive	pre	3.18	0.09	40	3.01	3.35
	post	2.93	0.10	40	2.73	3.13
Negative	pre	1.27	0.04	40	1.19	1.35
	post	1.17	0.04	40	1.09	1.24

Model 1: Ratings of funniness

Formula: humor_rating ~ feedback * condition + (1 + condition + feedback |subject) + (1|JokeID)

Information on the data included in the model:

- Jokes only

Table S19:
Results (Experiment 3): Humor rating as a function of condition and feedback

	β	SE	t	CI_l	CI_u	$Stab_{min}$	$Stab_{max}$	p_{Satter}	$R^2_{partial}$	CI_{lR2}	CI_{uR2}	LRT:Model	χ^2	df	p
(Intercept)	1.94	0.09	20.68	1.75	2.11	1.91	1.96	.000	-	-	-	(Intercept)	-	-	-
feedback_laughter	0.31	0.06	4.88	0.19	0.42	0.29	0.33	.000	.0101	.0038	.0194	feedback	27.42	1	<.001
condition_unconstrained	0.14	0.07	2.04	-0.01	0.26	0.11	0.16	.045	.0020	.0000	.0070	condition	6.7	1	.010
feedback_laughter:condition_unconstrained	0.03	0.07	0.41	-0.11	0.17	-0.01	0.06	.684	.0000	.0000	.0023	feedback:condition	0.17	1	.685

Notes: β = model estimate, SE = standard error of the estimate, CI = lower and upper 95% bootstrapped confidence intervals, Stab = estimate ranges leaving out one participant at a time, p_{Satter} = Satterwhite approximated p-values, $R^2_{partial}$ = partial R^2 with lower and upper confidence intervals, LRT = Likelihood ratio test. The model's overall $R^2_{partial}$ was: .0265 [.0162,.0412]

Figure S13:
Model-predicted humor rating by condition and feedback.

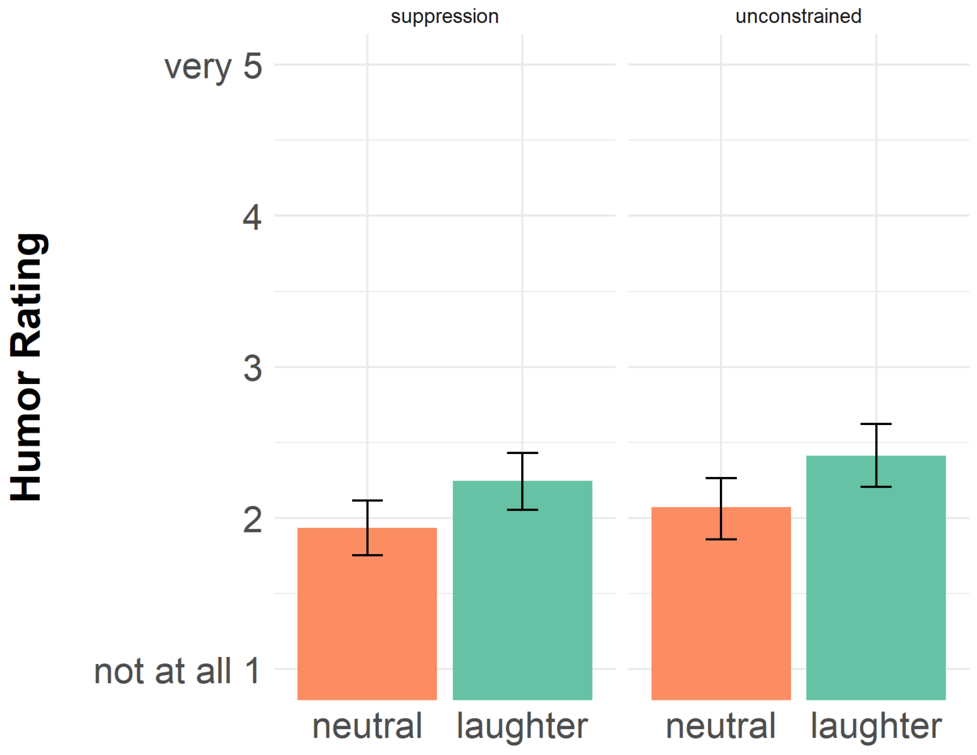

Notes: The error bars show the 95% bootstrapped CI of the model predictions

Model 2: Muscle Inhibition modulated by Social Feedback

Formula: smile index (BS) ~ Feedback * segments + (1+ Feedback * segments|subject) + (1+Feedback * segments|jokeID)

Information on the data included in the model:

- suppression condition and joke trials only
- data were baseline corrected before joke onset
- reference levels are neutral
- Index was calculated based on the within-subject z-transformed muscle.

Table S20:

Results of Model 2 (Experiment 3): Smile index as a function of social feedback over time

	β	SE	t	CI_l	CI_u	$Stab_{min}$	$Stab_{max}$	p_{Satter}	$R^2_{partial}$	CI_{lR2}	CI_{uR2}	LRT:Model	χ^2	df	p
(Intercept)	-0.17	0.08	-2.04	-0.32	-0.01	-0.20	-0.14	.046	-	-	-	(Intercept)	-	-	-
Feedback_Laughter	0.67	0.11	6.37	0.47	0.88	0.62	0.69	.000	.0803	.0689	.0925	Feedback	30.3	1	<.001
Segment_z	0.00	0.02	-0.24	-0.04	0.03	-0.01	0.00	.812	.0000	.0000	.0007	Segment	6.28	1	.012
Feedback_Laughter:Segment_z	0.09	0.03	2.62	0.03	0.16	0.08	0.10	.011	.0015	.0003	.0038	Feedback:Segment	6.86	1	.009

Notes: β = model estimate, SE = standard error of the estimate, CI = lower and upper 95% bootstrapped confidence intervals, Stab = estimate ranges leaving out one participant at a time, p_{Satter} = Satterwhite approximated p-values, $R^2_{partial}$ = partial R^2 with lower and upper confidence intervals, LRT = Likelihood ratio test. The model's overall $R^2_{partial}$ was: .0825 [.0712,.0950]

Figure S14:

Model 2: Smile Index by Social Feedback (Suppression Trials, Jokes).

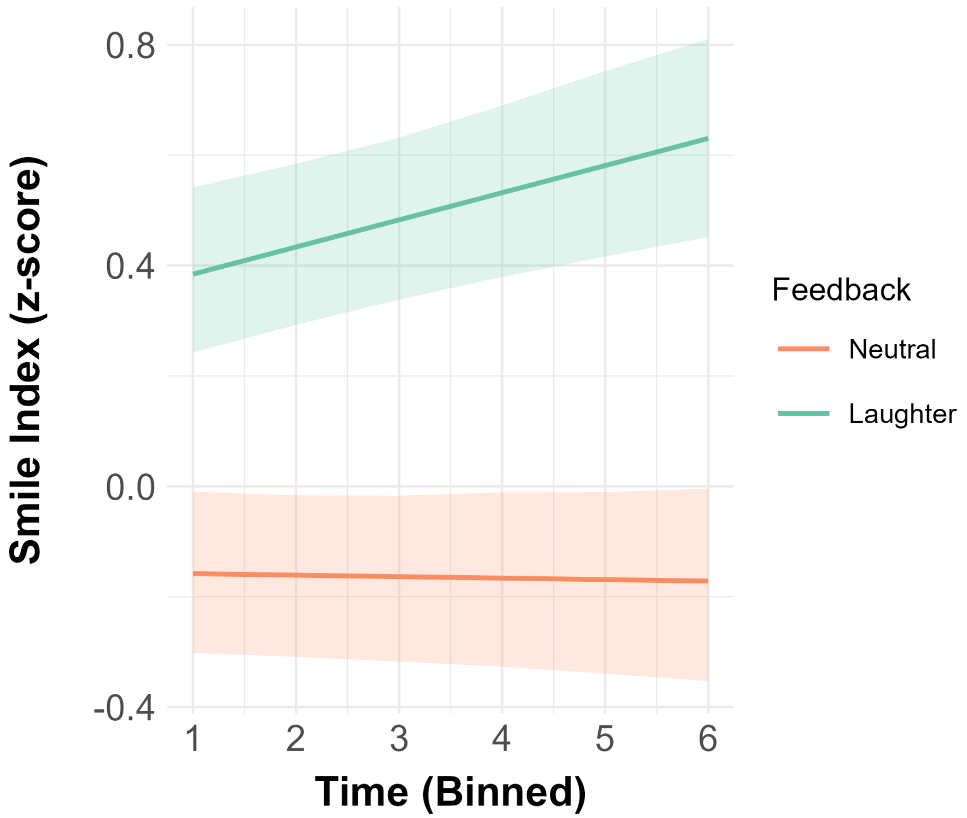

Notes: The shaded areas show the 95% bootstrapped CI of the model predictions

Model 3: Influence of Humorous Stimuli on Mimicry Suppression

Formula: smile index (BS) ~ JorF * Segment_z + (1+JorF*Segment_z|Subject)

Information on the data set:

- laughter feedback trials of the suppression condition only
- data were baseline corrected before feedback
- reference levels are "fact"
- Index was calculated based on the within-subject z-transformed muscle.

Table S21:
Results of Model 3 (Experiment 3): Smile index as a function of joke or facts over time

	β	SE	t	CI_l	CI_u	$Stab_{min}$	$Stab_{max}$	p_{Satter}	$R^2_{partial}$	CI_{lR2}	CI_{uR2}	LRT:Model	χ^2	df	p
(Intercept)	-0.48	0.13	-3.84	-0.71	-0.23	-0.53	-0.42	.000	-	-	-	(Intercept)	-	-	-
JorF_Joke	0.55	0.16	3.32	0.22	0.84	0.47	0.62	.002	.0135	.0078	.0209	JorF	9.55	1	.002
Segment_z	0.21	0.06	3.44	0.10	0.34	0.19	0.23	.001	.0028	.0006	.0066	Segment	18.04	1	<.001
JorF_Joke:Segment_z	-0.05	0.07	-0.81	-0.19	0.08	-0.07	-0.03	.419	.0001	.0000	.0016	JorF:Segment	0.46	1	.496

Notes: β = model estimate, SE = standard error of the estimate, CI = lower and upper 95% bootstrapped confidence intervals, Stab = estimate ranges leaving out one participant at a time, p_{Satter} = Satterwhite approximated p-values, $R^2_{partial}$ = partial R^2 with lower and upper confidence intervals, LRT = Likelihood ratio test. The model's overall $R^2_{partial}$ was: .0204 [.0136,.0296]

Figure S15:
Model 3: Smile Index for Jokes and Facts (Suppression Trials, Laughter Feedback).

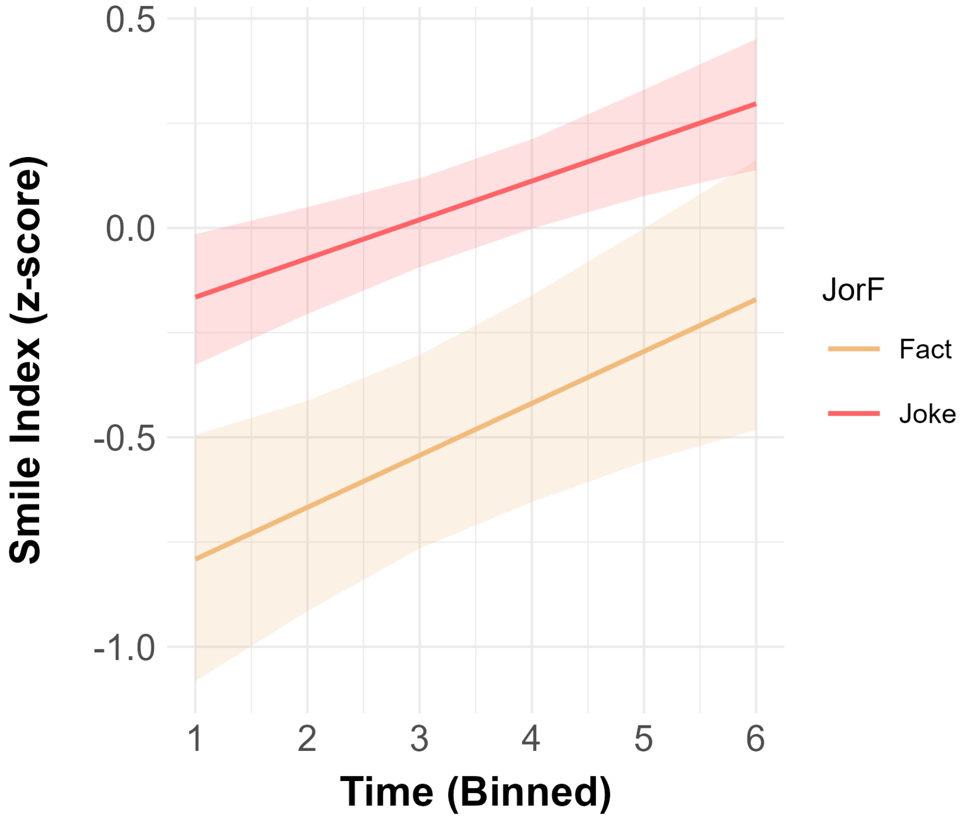

Notes: The shaded areas show the 95% bootstrapped CI of the model predictions
